# Variation in parental experiences with their child’s hospitalization over the COVID-19 pandemic

**DOI:** 10.1186/s41687-023-00626-3

**Published:** 2023-11-10

**Authors:** Kyle A. Kemp, Paul Fairie, Brian Steele, Maria J. Santana

**Affiliations:** 1 Department of Community Health Sciences, University of Calgary, TRW Building, 5th Floor 3280 Hospital Drive NW, Calgary, AB T2N 4Z6 Canada; 2 Patient Engagement Platform, Alberta Strategy for Patient-Oriented Research (SPOR), Calgary, AB Canada; 3 Department of Paediatrics, University of Calgary, Calgary, AB Canada; 4 School of Public Health, University of Alberta, Edmonton, AB Canada

**Keywords:** Pediatrics, Hospitalization, Patient experience, PREM, Survey, COVID-19

## Abstract

**Background:**

Hospitals and healthcare workers have been greatly impacted by the COVID-19 pandemic. The potential impacts upon the patient experience have been less documented, particularly in the pediatric setting. Our aim was to examine how parental experiences with their child’s hospitalization varied during the COVID-19 pandemic at two children’s hospitals in Alberta, Canada.

**Methods:**

A random sample of parents were surveyed within six weeks of their child’s discharge from Alberta’s two children’s hospitals. Surveys were administered using the Alberta Pediatric Inpatient Experiences Survey (APIES) - a validated instrument used to assess parental experiences during their child’s hospitalization. Surveys were linked with administrative inpatient records. Three cohorts were created based on hospital discharge date: Pre-COVID (Pre: April 2019 to March 2020), COVID year one (C1: April 2020 to March 2021), and COVID year two (C2: April 2021 to March 2022). We examined 48 survey questions, including four overall rating scales. Survey responses were Likert scales. These were transformed to normalized scores from 0 (worst) to 100 (best). Differences between cohorts were assessed using ANOVA and the post-hoc Tukey test.

**Results:**

A total of 3,611 surveys (1,314 Pre; 997 C1; 1,300 C2) were completed over the three-year period. Five questions showed differences between the Pre and C1 periods, six showed differences between Pre and C2, and 13 showed differences between C1 and C2. Among these questions, scores pre-COVID were lower than COVID year one, while results in COVID year two were lower than pre-COVID and COVID year one. Thirty-one survey questions showed no statistical differences between the three time periods. For the overall ratings, only hospital rating showed a difference in any of the periods (91.4 C1 vs. 90.2 C2). Overall ratings of doctors, nurses, and recommendation of the hospital to others showed no differences.

**Conclusion:**

This study showed that the experiences of parents during the first year of the COVID-19 pandemic were mildly better or comparable to historical results. This changed over the following year, where lower scores were reported on 13 questions.

**Supplementary Information:**

The online version contains supplementary material available at 10.1186/s41687-023-00626-3.

## Introduction

Approximately three years after the first cases were detected, the COVID-19 pandemic continues to have lasting effects upon health systems, healthcare providers and the general public. To date, over 622 thousand cases of the virus, and over 5,300 deaths have been recorded in Alberta, Canada [1]; a province of approximately 4.4 million residents. At the outset of the virus, significant public health restrictions were implemented across the province, resulting in disruptions to healthcare service delivery. These included the postponement of scheduled surgeries, alterations/removal of hospital visitation opportunities, and a significant shift of in-person consultation to virtual care [2]. Historic reductions in emergency department visit and inpatient volumes were also observed [3–5].

Many hospitals and health systems have adopted the Triple Aim framework as a “north star” in their approach to provide high quality care. The aims of the Triple Aim are to improve the health of populations, reduce per capita costs, and improve the patient care experience [6]. In Alberta, healthcare services are provided by Alberta Health Services (AHS); the province’s single health authority. AHS has adopted a fourth aim – to improve the provider experience [7]. With respect to COVID-19, its associated impacts upon the health of populations, healthcare costs, and the provider experience have been well-documented. These include topics such as the observed decrease in life expectancy [8], and the high costs of care, which have been primarily driven by the high cost of ICU care required for COVID patients [9]. From a workforce perspective, stress, moral hazard, burnout, and an increased number of healthcare professionals who have intentions to leave their profession have been documented [10–12].

The experiences of patients have also been documented, but primarily within the context of virtual services, or the risk/fears of contracting COVID among certain populations (e.g., those who are immunocompromised) [13, 14]. During the pandemic, however, there have been many who have continued to require acute (e.g., inpatient) care. Their experiences have been less documented. Recently, our team published a paper which explored the experiences of adults who were hospitalized across Alberta during the first year of the COVID pandemic [15]. Our results were similar to from similar studies in the United States [16] and the United Kingdom [17].

To date, reports of patient and parent experiences with pediatric inpatient hospitalization during COVID-19 have been scant. Therefore, our aim was to explore these experiences in detail, using a validated survey at Alberta’s two children’s hospitals.

## Methods

### Study Population

This study examined the experiences of parents’ whose child was hospitalized over the course of a three-year period (April 2019 to March 2022) in Alberta’s two stand-alone children’s hospitals (Alberta Children’s [Calgary], Stollery Children’s [Edmonton]). To compare potential differences between pre-COVID and during the COVID-19 pandemic, three cohorts were constructed based upon the child’s discharge date from hospital. The Pre-COVID cohort included discharges from April 2019 to March 2020. The COVID Year One (C1) cohort included discharges from April 2020 to March 2021, while the COVID Year Two (C2) cohort encompassed discharges from April 2021 to March 2022.

### Survey Instrument and Protocol

Parent experiences with their child’s hospital care were captured using the Alberta Pediatric Inpatient Experiences Survey (APIES). The APIES is a validated instrument which is based upon the Child Hospital Consumer Assessment of Healthcare Providers and Systems (Child HCAHPS) survey [18, 19]. In the province of Alberta, Canada, Alberta Health Services (AHS) is the sole provider of pediatric inpatient care. The APIES is administered by telephone by AHS within 2 and 42 days of the child’s discharge from hospital. Respondents (parents and guardians) are randomly selected, and contacted between 9AM and 9PM on weekdays, and 9AM and 4PM on weekends. Surveys are conducted by a team trained of interviewers, using a standard script and answers to frequently asked questions. In a typical year, approximately 2,500 surveys are captured from 14 hospitals (2 children’s, 12 primarily adult sites) across the province. To be eligible to participate, the respondent’s child must be less than 18 years old at the time of hospital discharge, have had an inpatient stay of at least 24 h, and be alive at the time of discharge. Care episodes limited to an emergency department visit, pertaining to healthy newborns (e.g., length of stay less 2 days), or taking place in a mental health unit are excluded from sampling as per AHS protocol.

Since it’s inception in October 2015, the APIES has had a response rate (e.g., percent of respondents who are contacted who complete the survey) of approximately 65%. The APIES contains 66 questions and takes approximately 15–20 min to administer. The survey asks respondents about multiple aspects of their child’s hospital care including communication with nurses and doctors, attention to safety and comfort, the physical environment, information sharing, and discharge planning and coordination. The APIES also contains four overall rating questions which ask respondents to provide a rating of the hospital, doctors, nurses, and their willingness to recommend the hospital to family members/friends. Responses to each survey question are Likert-type scales (e.g., always, usually, sometimes, never), and overall rating questions have response options ranging from 0 (worst possible) to 10 (best possible). The APIES concludes with a series of demographic questions. These include respondent age, level of educational attainment, relationship to the child, amount of time spent with the child during the hospital stay, and rating of the child’s health.

### Data linkage

Each completed survey was linked to the corresponding inpatient record from the Discharge Abstract Database (DAD) [20]. The DAD contains records from all inpatient discharges in Alberta. The data set is a source of demographic and clinical data, including patient sex, age, hospital, and length of stay; variables which were extracted for this study. Demographic and clinical variables which were retained for analysis included sex of the patient, patient age group (under 1 year, 1–4 years, 5–8 years, 9–12 years, 13–17 years), respondent relationship with the child (mother, father, other), respondent education level (8th grade or less, some high school, high school or equivalent, college or other certificate/diploma, undergraduate, post-graduate or professional degree), parent-reported health status of the child (excellent, very good, good, fair, poor), respondent time spent at hospital with child (all/nearly all of the time, most of the time, some of the time, little of the time, none), and length of hospital stay (less than 3 days, 3–7 days, more than 7 days). physical health, and self-reported mental/emotional health (both reported as excellent, very good, good, fair, or poor).

### Analysis

We analyzed the results from 47 survey questions. The 19 questions not included in our analyses were screener questions, demographic questions, and one open-ended question which asks respondents if there was anything else that they would like to share about their child’s hospital stay. Descriptive statistics (frequencies, percentages) were generated for the demographic and clinical variables, and chi-square tests were used to compare differences between these among the three study cohorts.

Responses to each survey question were normalized on a scale of 0 (worst) to 100 (best). For example, Likert scales were converted as 100 = always, 66.66 = usually, 33.33 = sometimes, and 0 = never. Overall rating scores (from 0 to 10) were multiplied by a factor of 10 (1 = 10, 2 = 20, etc.). Mean differences in scores for each survey question were assessed using ANOVA, while differences between groups were assessed using post-hoc Tukey tests. Effect size was calculated using Cohen’s d [21]. Values of 0.2 or less were deemed as a small effect, 0.21 to 0.5 as moderate, and greater than 0.5 as large [21]. All analyses were performed using SAS 9.4 for Windows (Cary, NC). A p-value of less than 0.05 was deemed statistically significant.

## Results

A total of 3,611 surveys (1,314 Pre-COVID, 997 COVID Year One, 1,300 COVID Year Two) were completed and linked with clinical data. The demographic and clinical characteristics of the three cohorts are presented in Table 1. Age of the child, age of the respondent, and length of stay were the only variables which showed significant differences between the time periods. Patient age (p < 0.01) and respondent age (p < 0.01) tended to be older over the course of COVID-19, while hospital length of stay among respondents tended to be shorter (p < 0.01). No differences were observed for sex of the patient, parent-reported health status of the child, respondent relationship with the child, respondent education level, and time that the respondent spent with the child while in hospital.

**Table 1:** Sample Demographics and Clinical Characteristics (n (%))

Variable	Pre-COVID	COVID Year 1	COVID Year 2	p
*Sex of Child (n=3,611)*				
Male	720 (54.8)	546 (54.8)	704 (54.2)	0.94
Female	594 (45.2)	451 (45.2)	596 (45.8)	
*Age of Child (n=3,611)*				
Under 1 year	376 (28.6)	251 (25.2)	306 (23.5)	<0.01
1 to 4 years	361 (27.5)	221 (22.2)	325 (25.0)	
5 to 8 years	208 (15.8)	140 (14.0)	158 (12.2)	
9 to 12 years	131 (10.0)	160 (16.1)	175 (13.5)	
13 to 17 years	238 (18.1)	225 (22.6)	336 (25.9)	
*Health Status of Child (n=3,566)*				
Excellent	437 (33.8)	314 (31.9)	399 (31.0)	0.11
Very good	349 (27.0)	284 (28.9)	410 (31.8)	
Good	291 (22.5)	228 (23.2)	249 (19.3)	
Fair	148 (11.4)	112 (11.4)	160 (12.4)	
Poor	69 (5.3)	46 (4.7)	70 (5.4)	
*Respondent Relationship (n=3,609)*				
Mother	1,102 (83.4)	819 (81.9)	1,053 (81.1)	0.44
Father	165 (12.6)	139 (14.0)	193 (14.8)	
Other(e.g. grandparent)	47 (3.6)	41 (4.1)	53 (4.1)	
*Age of Respondent (n=3,588)*				
Less than 25 years	47 (3.6)	19 (1.9)	47 (3.6)	<0.01
25 to 34 years	498 (38.1)	337 (33.9)	405 (31.2)	
35 to 44 years	569 (43.5)	436 (43.9)	566 (43.6)	
45 years or older	194 (14.8)	201 (20.2)	279 (21.5)	
*Education Level of Respondent (n=3,536)*				
8^th^ Grade or less	16 (1.3)	12 (1.2)	13 (1.0)	0.7
Some high school	67 (5.2)	33 (3.4)	59 (4.6)	
High school or equivalent	196 (15.3)	162 (16.6)	196 (15.3)	
College or other certificate/diploma	416 (32.5)	329 (33.7)	405 (31.6)	
Undergraduate (some or complete)	327 (25.6)	253 (25.9)	338 (26.4)	
Post-graduate/professional degree	258 (20.2)	187 (18.2)	269 (21.0)	
*Time Spent at Hospital with Child (n=3,607)*				
All/nearly all the time	999 (76.0)	739 (74.3)	933 (71.9)	0.06
Most of the time	256 (19.5)	189 (19.0)	271 (20.9)	
Some of the time	49 (3.7)	48 (4.8)	72 (5.6)	
A little of the time	7 (0.5)	13 (1.3)	12 (0.9)	
None of the time	2 (0.2)	6 (0.6)	10 (0.8)	
*Length of Child’s Hospital Stay (n=3,611)*				
Less than 3 days	526 (40.0)	463 (46.4)	620 (47.8)	<0.01
3 to 7 days	497 (37.8)	353 (35.4)	462 (35.5)	
Longer than 7 days	291 (22.2)	181 (18.2)	218 (16.8)	

Note: The number of responses is included for each variable, as some respondents did not respond to all questions

The normalized scores for each survey question that we examined are presented in Table 2. Results are presented for each of the three cohorts. Five survey questions showed significant differences between the Pre-COVID and C1 cohorts. These included doctors listening to parents (90.7 vs. 93.0), being asked about current medications (77.3 vs. 72.6), providers working together to provide care (90.0 vs. 92.3), checking child’s wristband/identification (81.9 vs. 85.9), and having things available for the child (80.3 vs. 67.7). Similar results were observed between the Pre-COVID and C2 cohorts, with six questions showing differences. Finally, 13 questions showed a significant difference between the C1 and C2 groups. Among these, results were consistently lower among the Pre-COVID group, when compared with the C1 group. Conversely, results for the C2 cohort were lower than both the pre-COVID and C1 groups. All six of the questions pertaining to communication between parents and healthcare providers (doctors, nurses) showed variation over the three-year period, with a decrease in scores of 0.6 to 2.3 observed. Other questions which showed variation were around information/communication of medication, tests, coordination of staff, the physical environment (e.g., cleanliness, quietness), and discharge planning. With respect to the overall rating questions, only hospital rating showed a difference in any of the periods (91.4 C1 vs. 90.2 C2). The overall rating of doctors and nurses, as well as the recommendation of the hospital to others showed no differences. Thirty-one survey questions showed no statistically significant differences between the three time periods.

**Table 2:** Normalized Score Results, all questions (0 = worst, 100 = best)

Question Wording	Pre-COVID	COVID Year 1	COVID Year 2	Sig. Result(s) *	Cohen’s d **
While your child was in this hospital’s Emergency Room, were you kept informed about what was being done for your child?	95.8	96.2	94.6		0.03, 0.06, 0.08
During this hospital stay, how often did your child’s nurses listen carefully to your child?	93.4	92.9	91.4		0.03, 0.12, 0.09
During this hospital stay, how often did your child’s nurses explain things in a way that was easy for your child to understand?	91.2	90.6	89.8		0.03, 0.07, 0.04
During this hospital stay, how often did your child’s nurses encourage your child to ask questions?	78.3	79.1	75.1		0.03, 0.11, 0.13
During this hospital stay, how often did your child’s doctors listen carefully to your child?	86.0	88.9	87.5		0.12, 0.06, 0.06
During this hospital stay, how often did your child’s doctors explain things in a way that was easy for your child to understand?	83.0	85.3	82.5		0.09, 0.01, 0.11
During this hospital stay, how often did your child’s doctors encourage your child to ask questions?	77.6	81.7	76.0	c	0.13, 0.05, 0.19
During this hospital stay, how often did your child’s nurses listen carefully to you?	91.1	91.2	88.8	b, c	0.01, 0.12, 0.12
During this hospital stay, how often did your child’s nurses explain things to you in a way that was easy to understand?	92.7	93.7	91.4	c	0.06, 0.07, 0.13
During this hospital stay, how often did your child’s nurses treat you with courtesy and respect?	94.6	95.9	94.0	c	0.09, 0.04, 0.13
During this hospital stay, how often did your child’s doctors listen carefully to you?	90.7	93.0	90.0	a, c	0.12, 0.03, 0.16
During this hospital stay, how often did your child’s doctors explain things to you in a way that was easy to understand?	91.9	93.1	90.9	c	0.07, 0.05, 0.12
During this hospital stay, how often did your child’s doctors treat you with courtesy and respect?	95.8	96.8	94.8	c	0.07, 0.07, 0.14
During this hospital stay, how often were you given as much privacy as you wanted when discussing your child’s care with providers?	89.0	90.3	89.4		0.06, 0.02, 0.04
Things that a family might know best about a child include how the child usually acts, what makes the child comfortable, and how to calm the child’s fears. During this hospital stay, did providers ask you about these types of things?	72.3	73.3	71.0		0.03, 0.04, 0.07
During this hospital stay, how often did providers talk with and act toward your child in a way that was right for your child’s age?	89.5	89.9	89.2		0.02, 0.01, 0.03
During this hospital stay, how often did healthcare providers introduce themselves and explain their roles?	90.5	91.1	89.6		0.03, 0.04, 0.08
During this hospital stay, how often did healthcare providers have a good understanding of your child’s condition and/or medical history?	82.7	84.0	82.0		0.05, 0.03, 0.08
During this hospital stay, how often did healthcare providers follow up on your concerns and observations?	85.2	87.2	84.9		0.09, 0.01, 0.10
During the first day of this hospital stay, were you asked to list or review all of the prescription medicines your child was taking at home?	89.5	87.4	89.9		0.07, 0.01, 0.09
During the first day of this hospital stay, were you asked to list or review all of the vitamins, herbal medicines, and over the counter medicines your child was taking at home?	77.3	72.6	75.8	a	0.12, 0.04, 0.09
During this hospital stay, how often would you say that the healthcare providers worked together to give the healthcare your child needed?	90.0	92.3	89.8	a, c	0.12, 0.01, 0.13
During this hospital stay, how often did healthcare providers keep you informed about what was being done for your child?	90.2	90.8	89.4		0.03, 0.04, 0.07
How often did providers give you as much information as you wanted about the results of these tests?	85.0	86.3	83.6	c	0.05, 0.06, 0.11
After pressing the call button, how often was help given as soon as you or your child wanted it?	86.6	88.5	86.5		0.09, 0.01, 0.10
Before giving your child any medicine, how often did providers or other hospital staff check your child’s wristband or confirm his or her identity in some other way?	81.9	85.9	84.7	a, b	0.15, 0.10, 0.05
During this hospital stay, did providers or other hospital staff tell you how to report if you had any concerns about mistakes in your child’s health care?	29.1	30.4	25.9	c	0.03, 0.11, 0.11
During this hospital stay, did providers or other hospital staff ask about your child’s pain as often as your child needed?	79.4	82.2	78.8		0.07, 0.01, 0.09
During this hospital stay, how often did the healthcare providers do everything they could to help your child with his or her pain?	91.4	91.3	89.9		0.01, 0.07, 0.07
During this hospital stay, did you feel you were appropriately involved in decision making about your child’s treatment/care?	89.7	90.4	89.5		0.03, 0.01, 0.04
During this hospital stay, did you have a clear understanding about your role in caring for your child?	93.5	94.3	93.8		0.04, 0.02, 0.03
During this hospital stay, how often were your child’s room and bathroom kept clean?	87.1	87.5	84.5	b, c	0.02, 0.11, 0.12
During this hospital stay, how often was the area around your child’s room quiet at night?	74.9	75.9	74.5	c	0.03, 0.02, 0.05
During this hospital stay, did the hospital have things available for your child that were right for your child’s age?	80.3	67.7	67.3	a, b	0.33, 0.34, 0.01
Before your child left the hospital, did a provider ask you if you had any concerns about whether your child was ready to leave?	89.5	89.3	86.6	b	0.01, 0.10, 0.10
Before your child left the hospital, did a provider talk with you as much as you wanted about how to care for your child’s health after leaving the hospital?	92.0	93.0	90.7	c	0.05, 0.06, 0.11
Before your child left the hospital, did a provider or hospital pharmacist explain in a way that was easy to understand how your child should take these new medicines after leaving the hospital?	89.4	89.9	91.0		0.02, 0.06, 0.04
Before your child left the hospital, did a provider or hospital pharmacist explain in a way that was easy to understand about possible side effects of these new medicines?	75.5	76.3	73.0		0.02, 0.07, 0.09
Before your child left the hospital, did a provider explain in a way that was easy to understand when your child could return to his or her regular activities?	82.7	82.5	81.1		0.01, 0.05, 0.04
Before your child left the hospital, did a provider explain in a way that was easy to understand what symptoms or health problems to look out for after your child left the hospital?	91.8	91.1	90.4		0.03, 0.06, 0.03
Before your child left the hospital, did you get information in writing about what symptoms or health problems to look out for after your child left the hospital?	78.2	81.6	80.3		0.09, 0.06, 0.04
During this hospital stay, how often did providers involve your child in discussions about his or her health care?	82.3	84.5	84.4		0.08, 0.08, 0.01
Before your child left the hospital, did a provider ask your child if he or she had any concerns about whether he or she was ready to leave?	78.4	85.0	82.9		0.19, 0.13, 0.07
Before your child left the hospital, did a provider talk to your child about how to take care of his or her health after leaving the hospital?	78.2	81.5	83.6		0.10, 0.17, 0.07
Using any number from 0 to 10, where 0 is the worst hospital possible and 10 is the best hospital possible, what number would you use to rate this hospital during your child’s stay? (8, 9, 10 Top Box)	90.8	91.4	90.2	c	0.05, 0.05, 0.10
Using any number from 0 to 10, where 0 is the worst possible nursing care and 10 is the best possible nursing care, what number would you give the care your child got from the nurses who treated him or her?	91.4	92.3	91.8		0.07, 0.03, 0.03
Using any number from 0 to 10 where 0 is the worst possible care and 10 is the best possible care, what number would you give the care your child got from all the doctors who treated him or her?	92.4	93.4	92.4		0.08, 0.00, 0.08
Would you recommend this hospital to your friends and family?	97.1	97.9	97.1		0.07, 0.00, 0.07

* Sig. Result(s): a: Pre-COVID vs. COVID Year 1; b: Pre-COVID vs. COVID Year 2; c: COVID Year 1 vs. COVID Year 2

** Cohen’s d reported as Pre-COVID vs. COVID Year 1, Pre-COVID vs. COVID Year 2, COVID Year 1 vs. COVID Year 2

## Discussion

We examined the experiences of parents whose children were hospitalized across Alberta, Canada during the COVID-19 pandemic. We explored the results prior to COVID-19, as well as during the first and second years of the pandemic at Alberta’s two children’s hospitals.

Over the course of the COVID-19 pandemic, respondents’ reports of their child’s inpatient care were largely positive. High scores were observed for most questions on the survey, with a few notable exceptions. For example, the question regarding parents being asked about how the child usually acts, what makes the child comfortable, and how to calm the child’s fears only received a score of 73.3 and 71.0 in COVID years one and two, respectively. However, these scores were not significantly different from the pre-pandemic score (72.3), suggesting that this may be an area for improvement at the two children’s hospitals studied. Another question which received a low score was the one pertaining to parents being told how to report any concerns about mistakes in their child’s health care (29.1 Pre-COVID, 30.4 C1, 25.9 C2; p < 0.05 for difference between C1 and C2). The question pertaining to parents being told about the potential side effects of medications is also a potential area for improvement (scores ranging from 73.0 to 76.2). This result has been highlighted in our previous work with child [22, 23] and adult [15, 24] inpatient surveys. Despite this, all four overall rating questions on the survey (hospital rating, nurse rating, doctor rating, willingness to recommend) scored 90 and above for all three cohorts/time periods.

In comparing the pre-COVID, COVID year one, and COVID year two cohorts, five survey questions showed significant differences between the Pre-COVID and C1 cohorts. Six questions showed significant differences between the Pre-COVID and C2 cohorts, and 13 questions showed a significant difference between the C1 and C2 groups. Of particular note, all six of the questions pertaining to communication between parents and healthcare providers (doctors, nurses) showed variation over the three-year period, with the lowest scores reported amongst the COVID year two cohort. Although we are unable to determine possible causes for this result, it is possibly due to staff burnout and the stresses of working in the prolonged period of heightened caution (e.g., enhanced infection control practices, use of extensive personal protective equipment, etc.) of the COVID pandemic.

Another interesting result was that of the physical environment. Lower hospital cleanliness and quietness scores were reported by the COVID year two cohort (84.5 and 74.5, respectively). This result is consistent with Riehm et al., who reported an increased number of nighttime room entries and sleep disruption among pediatric patients in Chicago at the outset of the COVID pandemic [25]. Interestingly, in our study, we did not see an immediate drop in scores at the outset of the pandemic, but much later (into year two). One potential reason for this may be the immediate decrease in hospital occupancy levels which were seen across our province in the first months of the pandemic [4]. In preparation for a potential influx of COVID-positive patients, many beds were left unoccupied, which may have also increased the amount of single-patient rooms, thus reducing the potential for nighttime disturbances. Other physical elements of hospital care also showed variation over the course of our study period. On the survey question which asked responded about whether the hospital had things available for their child (e.g., books, toys, games) that were right for their child’s age, scores had a sharp decrease; from 80.3 pre-COVID to 67.7 in COVID year one, and 67.3 in COVID year two. This finding is not surprising given the increased emphasis placed upon infection control practices during the pandemic. Many, if not all items typically available to children who are hospitalized were removed during the course of the pandemic. Parents may have also been hesitant to bring their own items during this time.

Despite our emphasis on potential areas for improvement, it is important to note that 31 of the 47 survey questions under study showed no differences across the three cohorts/time periods. These findings echo those of an Australian study which showed that the high quality of care was largely preserved (i.e., survey scores during COVID were similar to those seen pre-pandemic) among pediatric oncology patients [26]. Also of note, was the finding that the length of stay over the course of the pandemic was significantly shorter than children who were hospitalized pre-COVID. Although we did not investigate the reasons for this observation, we believe that this was associated with the desire to preserve low hospital occupancy as a means of preparation for a large influx of COVID patients. Future research is needed to confirm this.

With respect to study limitations, we reported on raw, normalized scores. Although we did compare selected demographic and clinical features across the three study cohorts, we did not do any risk adjustment, as is typically done using the “top box” method of reporting Child HCAHPS/HCAHPS results [27]. Second, as the APIES (like all surveys) is retrospective, there is always a potential for recall bias on the part of respondents [28]. To this end, AHS uses a standard survey protocol, which reminds respondents of the hospital discharge in question. Given the nature of our research agreement, were not able to assess the potential for non-response bias [29] as well as the number of respondents who completed multiple surveys (resulting from multiple hospitalizations) in our sample. Future studies could evaluate the demographic and clinical characteristics of respondents and non-respondents to the APIES, as has been done among the adult inpatient population by our team [30] and abroad [31]. Given that the APIES is only administered by telephone, our results may not be generalizable to other survey formats (e.g., mail, phone, e-mail). As the APIES is only administered in English, our results may also not apply to non-English speaking parents of children who are hospitalized in our province.

## Conclusion

This is one of the first studies to explore the experiences of parents of children who were hospitalized during COVID-19. Although the results to some questions showed a modest decrease in the second year of the pandemic, the majority of survey questions showed no difference in scores between pre-COVID results. Although our results do provide some opportunities for improvement, the underlying scores highlight the high quality of care that children and families received at Alberta’s two children’s hospitals throughout the pandemic. Future research is necessary to examine the potential for response shift in our results, as well as the possible associations of patient experience scores with other outcomes (e.g., hospital readmission, patient safety events).

### Electronic supplementary material

Below is the link to the electronic supplementary material.

Supplementary Material 1

## Data Availability

The data that support the findings of this study are available from Alberta Health Services but restrictions apply to the availability of these data, which were used under license for the current study, and so are not publicly available.
