# Supplementary material for: Variation in parental experiences with their child’s hospitalization over the COVID-19 pandemic
Source: J Patient Rep Outcomes. 2023 Nov 10;7:114. doi: 10.1186/s41687-023-00626-3 (PMC10638244; doi:10.1186/s41687-023-00626-3)
Supplement: Supplementary file 1 — Supplementary Material 1 [file 41687_2023_626_MOESM1_ESM.docx]

**Variation in Parental Experiences with their Child’s**

**Hospitalization over the COVID-19 Pandemic**

**ABSTRACT**

**Background:** Hospitals and healthcare workers have been greatly impacted by the COVID-19 pandemic. The potential impacts upon the patient experience have been less documented, particularly in the pediatric setting. Our aim was to examine how parental experiences with their child’s hospitalization varied during the COVID-19 pandemic at two children’s hospitals in Alberta, Canada.

**Methods:** A random sample of parents were surveyed within six weeks of their child’s discharge from Alberta’s two children’s hospitals. Surveys were administered using the Alberta Pediatric Inpatient Experiences Survey (APIES) - a validated instrument used to assess parental experiences during their child’s hospitalization. Surveys were linked with administrative inpatient records. Three cohorts were created based on hospital discharge date: Pre-COVID (Pre: April 2019 to March 2020), COVID year one (C1: April 2020 to March 2021), and COVID year two (C2: April 2021 to March 2022). We examined 48 survey questions, including four overall rating scales. Survey responses were Likert scales. These were transformed to normalized scores from 0 (worst) to 100 (best). Differences between cohorts were assessed using ANOVA and the post-hoc Tukey test.

**Results:** A total of 3,611 surveys (1,314 Pre; 997 C1; 1,300 C2) were completed over the three-year period. Five questions showed differences between the Pre and C1 periods, six showed differences between Pre and C2, and 13 showed differences between C1 and C2. Among these questions, scores pre-COVID were lower than COVID year one, while results in COVID year two were lower than pre-COVID and COVID year one. Thirty-one survey questions showed no statistical differences between the three time periods. For the overall ratings, only hospital rating showed a difference in any of the periods (91.4 C1 vs. 90.2 C2). Overall ratings of doctors, nurses, and recommendation of the hospital to others showed no differences.

**Conclusion:** This study showed that the experiences of parents during the first year of the COVID-19 pandemic were mildly better or comparable to historical results. This changed over the following year, where lower scores were reported on 13 questions.

**Keywords:** Pediatrics, hospitalization, patient experience, PREM, survey, COVID-19

**INTRODUCTION**

Approximately three years after the first cases were detected, the COVID-19 pandemic continues to have lasting effects upon health systems, healthcare providers and the general public. To date, over 622 thousand cases of the virus, and over 5,300 deaths have been recorded in Alberta, Canada [1]; a province of approximately 4.4 million residents. At the outset of the virus, significant public health restrictions were implemented across the province, resulting in disruptions to healthcare service delivery. These included the postponement of scheduled surgeries, alterations/removal of hospital visitation opportunities, and a significant shift of in-person consultation to virtual care [2]. Historic reductions in emergency department visit and inpatient volumes were also observed [3-5].

Many hospitals and health systems have adopted the Triple Aim framework as a “north star” in their approach to provide high quality care. The aims of the Triple Aim are to improve the health of populations, reduce per capita costs, and improve the patient care experience [6]. In Alberta, healthcare services are provided by Alberta Health Services (AHS); the province’s single health authority. AHS has adopted a fourth aim – to improve the provider experience [7]. With respect to COVID-19, its associated impacts upon the health of populations, healthcare costs, and the provider experience have been well-documented. These include topics such as the observed decrease in life expectancy [8], and the high costs of care, which have been primarily driven by the high cost of ICU care required for COVID patients [9]. From a workforce perspective, stress, moral hazard, burnout, and an increased number of healthcare professionals who have intentions to leave their profession have been documented [10-12].

The experiences of patients have also been documented, but primarily within the context of virtual services, or the risk/fears of contracting COVID among certain populations (e.g., those who are immunocompromised) [13, 14]. During the pandemic, however, there have been many who have continued to require acute (e.g., inpatient) care. Their experiences have been less documented. Recently, our team published a paper which explored the experiences of adults who were hospitalized across Alberta during the first year of the COVID pandemic [15]. Our results were similar to from similar studies in the United States [16] and the United Kingdom [17].

To date, reports of patient and parent experiences with pediatric inpatient hospitalization during COVID-19 have been scant. Therefore, our aim was to explore these experiences in detail, using a validated survey at Alberta’s two children’s hospitals.

**METHODS**

**Study Population**

**This study examined the experiences of parents’ whose child was hospitalized over the course of a three-year period (April 2019 to March 2022) in Alberta’s two stand-alone children’s hospitals (Alberta Children’s [Calgary], Stollery Children’s [Edmonton]). To compare potential differences between pre-COVID and during the COVID-19 pandemic, three cohorts were constructed based upon the child’s discharge date from hospital. The Pre-COVID cohort included discharges from April 2019 to March 2020. The COVID Year One (C1) cohort included discharges from April 2020 to March 2021, while the COVID Year Two (C2) cohort encompassed discharges from April 2021 to March 2022.**

**Survey Instrument and Protocol**

**Parent experiences with their child’s hospital care were captured using the** Alberta Pediatric Inpatient Experiences Survey (APIES). The APIES is a validated instrument which is based upon the Child Hospital Consumer Assessment of Healthcare Providers and Systems (Child HCAHPS) survey [18, 19]. In the province of Alberta, Canada, Alberta Health Services (AHS) is the sole provider of pediatric inpatient care. The APIES is administered by telephone by AHS within 2 and 42 days of the child’s discharge from hospital. Respondents (parents and guardians) are randomly selected, and contacted between 9AM and 9PM on weekdays, and 9AM and 4PM on weekends. Surveys are conducted by a team trained of interviewers, using a standard script and answers to frequently asked questions. In a typical year, approximately 2,500 surveys are captured from 14 hospitals (2 children’s, 12 primarily adult sites) across the province. To be eligible to participate, the respondent’s child must be less than 18 years old at the time of hospital discharge, have had an inpatient stay of at least 24 hours, and be alive at the time of discharge. Care episodes limited to an emergency department visit, pertaining to healthy newborns (e.g., length of stay less 2 days), or taking place in a mental health unit are excluded from sampling as per AHS protocol.

Since it’s inception in October 2015, the APIES has had a response rate (e.g., percent of respondents who are contacted who complete the survey) of approximately 65%. The APIES contains 66 questions and takes approximately 15-20 minutes to administer. The survey asks respondents about multiple aspects of their child’s hospital care including communication with nurses and doctors, attention to safety and comfort, the physical environment, information sharing, and discharge planning and coordination. The APIES also contains four overall rating questions which ask respondents to provide a rating of the hospital, doctors, nurses, and their willingness to recommend the hospital to family members/friends. Responses to each survey question are Likert-type scales (e.g., always, usually, sometimes, never), and overall rating questions have response options ranging from 0 (worst possible) to 10 (best possible). The APIES concludes with a series of demographic questions. These include respondent age, level of educational attainment, relationship to the child, amount of time spent with the child during the hospital stay, and rating of the child’s health.

**Data Linkage**

Each completed survey was linked to the corresponding inpatient record from the Discharge Abstract Database (DAD) [20]. The DAD contains records from all inpatient discharges in Alberta. The data set is a source of demographic and clinical data, including patient sex, age, hospital, and length of stay; variables which were extracted for this study. Demographic and clinical variables which were retained for analysis included sex of the patient, patient age group (under 1 year, 1-4 years, 5-8 years, 9-12 years, 13-17 years), respondent relationship with the child (mother, father, other), respondent education level (8^th^ grade or less, some high school, high school or equivalent, college or other certificate/diploma, undergraduate, post-graduate or professional degree), parent-reported health status of the child (excellent, very good, good, fair, poor), respondent time spent at hospital with child (all/nearly all of the time, most of the time, some of the time, little of the time, none), and length of hospital stay (less than 3 days, 3-7 days, more than 7 days). physical health, and self-reported mental/emotional health (both reported as excellent, very good, good, fair, or poor).

**Analysis**

We analyzed the results from 47 survey questions. The 19 questions not included in our analyses were screener questions, demographic questions, and one open-ended question which asks respondents if there was anything else that they would like to share about their child’s hospital stay. Descriptive statistics (frequencies, percentages) were generated for the demographic and clinical variables, and chi-square tests were used to compare differences between these among the three study cohorts.

Responses to each survey question were normalized on a scale of 0 (worst) to 100 (best). For example, Likert scales were converted as 100=always, 66.66=usually, 33.33=sometimes, and 0=never. Overall rating scores (from 0 to 10) were multiplied by a factor of 10 (1=10, 2=20, etc.). Mean differences in scores for each survey question were assessed using ANOVA, while differences between groups were assessed using post-hoc Tukey tests. Effect size was calculated using Cohen’s d [21]. Values of 0.2 or less were deemed as a small effect, 0.21 to 0.5 as moderate, and greater than 0.5 as large [21]. All analyses were performed using SAS 9.4 for Windows (Cary, NC). A p-value of less than 0.05 was deemed statistically significant.

**RESULTS**

A total of 3,611 surveys (1,314 Pre-COVID, 997 COVID Year One, 1,300 COVID Year Two) were completed and linked with clinical data. The demographic and clinical characteristics of the three cohorts are presented in Table 1. Age of the child, age of the respondent, and length of stay were the only variables which showed significant differences between the time periods. Patient age (p<0.01) and respondent age (p<0.01) tended to be older over the course of COVID-19, while hospital length of stay among respondents tended to be shorter (p<0.01). No differences were observed for sex of the patient, parent-reported health status of the child, respondent relationship with the child, respondent education level, and time that the respondent spent with the child while in hospital.

The normalized scores for each survey question that we examined are presented in Table 2. Results are presented for each of the three cohorts. Five survey questions showed significant differences between the Pre-COVID and C1 cohorts. These included doctors listening to parents (90.7 vs. 93.0), being asked about current medications (77.3 vs. 72.6), providers working together to provide care (90.0 vs. 92.3), checking child’s wristband/identification (81.9 vs. 85.9), and having things available for the child (80.3 vs. 67.7). Similar results were observed between the Pre-COVID and C2 cohorts, with six questions showing differences. Finally, 13 questions showed a significant difference between the C1 and C2 groups. Among these, results were consistently lower among the Pre-COVID group, when compared with the C1 group. Conversely, results for the C2 cohort were lower than both the pre-COVID and C1 groups. All six of the questions pertaining to communication between parents and healthcare providers (doctors, nurses) showed variation over the three-year period, with a decrease in scores of 0.6 to 2.3 observed. Other questions which showed variation were around information/communication of medication, tests, coordination of staff, the physical environment (e.g., cleanliness, quietness), and discharge planning. With respect to the overall rating questions, only hospital rating showed a difference in any of the periods (91.4 C1 vs. 90.2 C2). The overall rating of doctors and nurses, as well as the recommendation of the hospital to others showed no differences. Thirty-one survey questions showed no statistically significant differences between the three time periods.

**DISCUSSION**

We examined the experiences of parents whose children were hospitalized across Alberta, Canada during the COVID-19 pandemic. We explored the results prior to COVID-19, as well as during the first and second years of the pandemic at Alberta’s two children’s hospitals.

Over the course of the COVID-19 pandemic, respondents’ reports of their child’s inpatient care were largely positive. High scores were observed for most questions on the survey, with a few notable exceptions. For example, the question regarding parents being asked about how the child usually acts, what makes the child comfortable, and how to calm the child's fears only received a score of 73.3 and 71.0 in COVID years one and two, respectively. However, these scores were not significantly different from the pre-pandemic score (72.3), suggesting that this may be an area for improvement at the two children’s hospitals studied. Another question which received a low score was the one pertaining to parents being told how to report any concerns about mistakes in their child's health care (29.1 Pre-COVID, 30.4 C1, 25.9 C2; p<0.05 for difference between C1 and C2). The question pertaining to parents being told about the potential side effects of medications is also a potential area for improvement (scores ranging from 73.0 to 76.2). This result has been highlighted in our previous work with child [22, 23] and adult [15, 24] inpatient surveys. Despite this, all four overall rating questions on the survey (hospital rating, nurse rating, doctor rating, willingness to recommend) scored 90 and above for all three cohorts/time periods.

In comparing the pre-COVID, COVID year one, and COVID year two cohorts, five survey questions showed significant differences between the Pre-COVID and C1 cohorts. Six questions showed significant differences between the Pre-COVID and C2 cohorts, and 13 questions showed a significant difference between the C1 and C2 groups. Of particular note, all six of the questions pertaining to communication between parents and healthcare providers (doctors, nurses) showed variation over the three-year period, with the lowest scores reported amongst the COVID year two cohort. Although we are unable to determine possible causes for this result, it is possibly due to staff burnout and the stresses of working in the prolonged period of heightened caution (e.g., enhanced infection control practices, use of extensive personal protective equipment, etc.) of the COVID pandemic.

Another interesting result was that of the physical environment. Lower hospital cleanliness and quietness scores were reported by the COVID year two cohort (84.5 and 74.5, respectively). This result is consistent with Riehm et al., who reported an increased number of nighttime room entries and sleep disruption among pediatric patients in Chicago at the outset of the COVID pandemic [25]. Interestingly, in our study, we did not see an immediate drop in scores at the outset of the pandemic, but much later (into year two). One potential reason for this may be the immediate decrease in hospital occupancy levels which were seen across our province in the first months of the pandemic [4]. In preparation for a potential influx of COVID-positive patients, many beds were left unoccupied, which may have also increased the amount of single-patient rooms, thus reducing the potential for nighttime disturbances. Other physical elements of hospital care also showed variation over the course of our study period. On the survey question which asked responded about whether the hospital had things available for their child (e.g., books, toys, games) that were right for their child's age, scores had a sharp decrease; from 80.3 pre-COVID to 67.7 in COVID year one, and 67.3 in COVID year two. This finding is not surprising given the increased emphasis placed upon infection control practices during the pandemic. Many, if not all items typically available to children who are hospitalized were removed during the course of the pandemic. Parents may have also been hesitant to bring their own items during this time.

Despite our emphasis on potential areas for improvement, it is important to note that 31 of the 47 survey questions under study showed no differences across the three cohorts/time periods. These findings echo those of an Australian study which showed that the high quality of care was largely preserved (i.e., survey scores during COVID were similar to those seen pre-pandemic) among pediatric oncology patients [26]. Also of note, was the finding that the length of stay over the course of the pandemic was significantly shorter than children who were hospitalized pre-COVID. Although we did not investigate the reasons for this observation, we believe that this was associated with the desire to preserve low hospital occupancy as a means of preparation for a large influx of COVID patients. Future research is needed to confirm this.

With respect to study limitations, we reported on raw, normalized scores. Although we did compare selected demographic and clinical features across the three study cohorts, we did not do any risk adjustment, as is typically done using the “top box” method of reporting Child HCAHPS/HCAHPS results [27]. Second, as the APIES (like all surveys) is retrospective, there is always a potential for recall bias on the part of respondents [28]. To this end, AHS uses a standard survey protocol, which reminds respondents of the hospital discharge in question. Given the nature of our research agreement, were not able to assess the potential for non-response bias [29] as well as the number of respondents who completed multiple surveys (resulting from multiple hospitalizations) in our sample. Future studies could evaluate the demographic and clinical characteristics of respondents and non-respondents to the APIES, as has been done among the adult inpatient population by our team [30] and abroad [31]. Given that the APIES is only administered by telephone, our results may not be generalizable to other survey formats (e.g., mail, phone, e-mail). As the APIES is only administered in English, our results may also not apply to non-English speaking parents of children who are hospitalized in our province.

**CONCLUSION**

This is one of the first studies to explore the experiences of parents of children who were hospitalized during COVID-19. Although the results to some questions showed a modest decrease in the second year of the pandemic, the majority of survey questions showed no difference in scores between pre-COVID results. Although our results do provide some opportunities for improvement, the underlying scores highlight the high quality of care that children and families received at Alberta’s two children’s hospitals throughout the pandemic. Future research is necessary to examine the potential for response shift in our results, as well as the possible associations of patient experience scores with other outcomes (e.g., hospital readmission, patient safety events).

**REFERENCES**

1. Government of Alberta. COVID-19 Alberta statistics. https://www.alberta.ca/stats/covid-19-alberta-statistics.htm#data-export. Accessed 22 Dec 2022.
2. Canadian Institute for Health Information. Impact of COVID-19 on Canada’s health care systems. https://www.cihi.ca/en/covid-19-resources/impact-of-covid-19-on-canadas-health-care-systems. Accessed 22 Dec 2022.
3. Dudevich A, Frood J (2021) Impact of the COVID-19 pandemic on health system use in Canada. Healthc Q 24(2):12-14. https://doi.org/10.12927/hcq.2021.26552.
4. Health Quality Council of Alberta. FOCUS on healthcare: hospital occupancy. Accessed August 11, 2021. https://focus.hqca.ca/emergencydepartments/hospital-occupancy
5. Rennert-May E, Leal J, Thanh NX et al (2021) The impact of COVID-19 on hospital admissions and emergency department visits: a population-based study. PLoS One 16(6):e0252441. https://doi.org/10.1371/journal.pone.0252441.
6. Berwick DM, Nolan TW, Whittington J (2008) The triple aim: care, health, and cost. Health Aff (Millwood) 27(3):759-769. https://doi.org/10.1377/hlthaff.27.3.759.
7. Alberta Health Services. 2020-22 Health Plan and 2021-22 Business Plan. https://www.albertahealthservices.ca/assets/about/org/ahs-org-hpbp-2020-2022.pdf. Accessed 22 Dec 2022.
8. Kuehn BM (2022) COVID-19 cuts life expectancy in dozens of countries. JAMA 327(3):209. https://doi.org/10.1001/jama.2021.24595.
9. Canadian Institute for Health Information. COVID-19 hospitalization and emergency department statistics. https://www.cihi.ca/en/covid-19-hospitalization-and-emergency-department-statistics. Accessed 22 Dec 2022.
10. Vanhaecht K, Seys D, Bruyneel L, Cox B, Kaesemans G, Cloet M et al (2021) COVID-19 is having a destructive impact on health-care workers’ mental well-being. Int J Qual Health Care 33(1):mzaa158. https://doi.org/10.1093/intqhc/mzaa158.
11. Sexton JB, Adair KC, Proulx J, Profit J, Cui X, Bae J et al (2022) Emotional exhaustion among US health care workers before and during the COVID-19 pandemic, 2019-2021. JAMA Netw Open 5(9):e2232748. https://doi.org/10.1001/jamanetworkopen.2022.32748.

1. Statistics Canada. Experiences of health care workers during the COVID-19 pandemic, September to November 2021. https://www150.statcan.gc.ca/n1/daily-quotidien/220603/dq220603a-eng.htm. Accessed 22 Dec 2022.
2. Chu C, Nayyar D, Bhattacharyya O, Martin D, Agarwal P, Mukerji G (2022) Patient and provider experiences with virtual care in a large, ambulatory care hospital in Ontario, Canada during the COVID-19 pandemic: observational study. J Med Internet Res 24(10):e38604. https://doi.org/10.2196/38604.
3. Dowling M, Eicher M, Drury A (2022) Experiences of cancer care in COVID-19: a longitudinal qualitative study. Eur J Oncol Nurs. https://doi.org/10.1016/j.ejon.2022.102228.
4. Kemp KA, Fairie P, Steele BJ, Santana MJ (2022) Adult experiences with hospitalization in Alberta, Canada during the COVID-19 pandemic: a comparative cross-sectional study. J Patient Exp. https://doi.org/10.1177/23743735221077518.
5. Drapeaux A, Jenson JA, Fustino N (2021) The impact of COVID-19 on patient experience within a Midwest hospital system: a case study. J Patient Exp. https://doi.org/10.1177/23743735211065298.
6. Key T, Kulkarni A, Kandhari V, Jawad Z, Hughes A, Mohanty K (2021) The patient experience of inpatient care during the COVID-19 pandemic: exploring patient perceptions, communication, and quality of care at a university teaching hospital in the United Kingdom. J Patient Exp. https://doi.org/10.1177/2374373521997742.
7. Toomey SL, Zaslavsky AM, Elliott MN, Gallagher PM, Fowler FJ Jr, Klein DJ (2015) The development of a pediatric inpatient experience of care measure: the Child HCAHPS. Pediatrics 136(2):360-369. https://doi.org/10.1542/peds.2015-0966.
8. Toomey SL, Elliott MN, Zaslavsky AM, Klein DJ, Ndon S, Hardy S (2017) Variation in family experience of pediatric inpatient care as measured by Child HCAHPS. Pediatrics 139(4):e20163372. https://doi.org/10.1542/peds.2016-3372.
9. Canadian Institute for Health Information. Discharge Abstract Database (DAD) metadata. https://www.cihi.ca/en/discharge-abstract-database-metadata. Accessed 22 Dec 2022.
10. Cohen J. Statistical Power Analysis for the Behavioral Sciences. 1988. Hillsdale, NJ: Erlbaum.
11. Kemp KA, Ahmed S, Quan H, Johnson D, Santana MJ (2018) Family experiences of pediatric inpatient care in Alberta, Canada: results from the Child HCAHPS survey. Hosp Pediatr 8(6):338-344. https://doi.org/10.1542/hpeds.2017-0191.
12. Ahmed S, Kemp K, Johnson D, Quan H, Santana MJ (2019) Identifying areas for improvement in pediatric inpatient care using the Child HCAHPS survey. Pediatr Child Health 25(6):365-371. https://doi.org/10.1093/pch/pxz031.
13. Kemp KA, Quan H, Knudtson ML, Oddone Paolucci E, Santana MJ (2019) Patient-reported experiences with coronary artery bypass grafting and valve replacement. Can J Cardiol 35(10):1344-1352. https://doi.org/10.1016/j.cjca.2019.05.008.
14. Riehm JM, Arora VM, Tatineni S, Erondu A, Mozer CL, Cook DJ et al (2021) The impact of the COVID-19 pandemic on nighttime room entries and sleep disruptions for pediatric patients. Sleep Med 84:76-81. https://doi.org/10.1016/j.sleep.2021.05.020
15. McCarthy MC, Beamish J, Bauld CM, Marks IR, Williams T, Olsson CA et al (2022) Parent perceptions of pediatric oncology care during the COVID-19 pandemic: an Australian study. Pediatr Blood Cancer 69(2):e29400. https://doi.org/10.1002/pbc.29400.
16. Centers for Medicare and Medicaid Services. A note about HCAHPS “boxes”. https://www.hcahpsonline.org/en/summary-analyses/#NoteAboutBoxes. Accessed 22 Dec 2022.
17. Salzberg CA, Kahn CN III, Foster NE et al (2019) Modernizing the HCAHPS survey: recommendations from patient experience leaders. https://www.aha.org/system/files/media/file/2019/07/FAH-White-Paper-Report-v18-FINAL.pdf. Accessed 22 Dec 2022.
18. Elliott MN, Zaslavsky AM, Goldstein E et al (2009) Effects of survey mode, patient mix, and nonresponse on CAHPS® hospital survey scores. Health Serv Res 44(2 Pt 1):501-518. https://doi.org/10.1111/j.1475-6773.2008.00914.x.
19. Kemp KA, Chan N, McCormack B (2015) The Alberta inpatient hospital experience survey: representativeness of sample and initial findings. 8(3):1-10. https://doi.org/10.29115/SP-2015-0012.
20. Lee B, Hollenbeck-Pringle D, Goldman V, Biondi E, Alverson B (2019) Are caregivers who respond to the Child HCAHPS survey reflective of all hospitalized pediatric patients? Hosp Pediatr 9(3):162-169. https://doi.org/10.1542/hpeds.2018-0139.
